# Pleiotropic functions of TIMP-1 in patients with chronic kidney disease

**DOI:** 10.1007/s00018-014-1592-5

**Published:** 2014-03-05

**Authors:** Kinga Musiał, Danuta Zwolińska

**Affiliations:** Department of Pediatric Nephrology, Wrocław Medical University, Borowska 213, 50-556 Wrocław, Poland

Sir,

We have read with great interest a recent in-depth review by Christian Ries [1], giving a picture of multiple activities of a tissue inhibitor of metalloproteinases (TIMP)-1 and revealing its complex cytokine impact on various cell functions. The pleiotropic role of TIMP-1 results from both metalloproteinase (MMP)-dependent and MMP-independent actions, creating a tool for the control of such diverse phenomena as apoptosis, cell differentiation or uncontrolled cell migration, resulting in metastases. In addition to a detailed theoretical background, the author has described the clinical implications of TIMP-1 activity, including cancer and cardiovascular disease. In our opinion, chronic kidney disease (CKD) is also worth mentioning among those examples.

CKD, with its irreversible progressive clinical course and multiple complications, seems an ideal example of TIMP-1 multifaceted activity, influencing chronic inflammation, impaired immunity, enhanced apoptosis and cardiovascular comorbidity. Our recent investigation has concentrated on various aspects of MMP and the role of TIMP-1 in the course of CKD in the pediatric population. We have shown that the increase of serum TIMP-1 concentrations in CKD children appears only in the late stages of renal failure and is delayed in comparison to the early rise of MMP values, being an anti-fibrotic response to extracellular matrix accumulation [2]. Moreover, TIMP-1 was the best predictor of TGFbeta1, a hallmark of fibrosis, a fact that is concordant with the tight TIMP–MMP–TGFbeta1 connections described by Ries [1].

Another interesting aspect of TIMP-1 activity is its anti-apoptotic function, realized by both direct, receptor-mediated, and indirect, by MMP inhibition, pathways [1]. Enhanced apoptosis is one of the key elements in the course of chronic kidney disease, additionally aggravated by the commencement of dialysis. Indeed, our investigation has revealed increased MMP/TIMP concentrations in chronically dialyzed children versus controls [3]. The additional discrepancy between dialysis modalities, most evident in the case of TIMP-1, has strengthened the suggestion that hemodialysis triggers apoptosis and reciprocal anti-apoptotic protection to greater extent than peritoneal dialysis. We have also noticed the statistically significant correlations between markers of matrix turnover and apoptosis [3]. In addition, regression analysis has shown that TIMP-1 was more accurate than TIMP-2, MMP-2, or MMP-9 in the prediction of the well-established apoptotic markers, sFas and sFasL [3].

The abovementioned MMP overactivity and MMP/TIMP imbalance disrupts the integrity of the extracellular matrix in the course of CKD. The subsequent tissue remodeling, together with cell damage and matrix accumulation, gives way to atherosclerosis, renal fibrosis and aggravated cell migration to sites of inflammation. In particular, cells losing their anchorage due to proteolytic overactivity, if not eliminated by anoikis, gain the ability for uncontrolled migration and further metastatic spread. The hallmark of anoikis activity is E-cadherin, released in a circulating form from destroyed cell–cell junctions, and easing epithelial-to-mesenchymal transition (EMT). We have shown for the first time significant correlations between this marker and MMP/TIMP in CKD children, particularly in those on chronic dialysis [4]. Moreover, on regression analysis, TIMP-1 has turned out to be the best predictor of E-cadherin among examined MMPs, TIMPs, and apoptotic indexes [4].

The last intriguing aspect of TIMP-1 activity in the CKD population is its functional similarity with heat shock proteins (HSP). HSP are classified as damage-associated molecular patterns (DAMP), highly conserved in their structure throughout species. Likewise, TIMPs appear in all mammals [1]. They all influence the immune system by activating (HSP) or inhibiting (TIMP-1) nuclear factor (NF)κB. We have revealed for the first time the significant correlations between HSP and MMP/TIMP both in pre-dialysis children and in those on chronic dialysis [2, 5]. Moreover, we discovered that TIMP-1 was the most valuable predictor of various HSP (Hsp27, Hsp90alpha, anti-Hsp60), suggesting that they share some common features, and that TIMP-1 may, under the circumstances, behave like a stress protein. However, this hypothesis requires further in-depth investigation.
